# Labeling of Anti-MUC-1 Binding Single Chain Fv Fragments to Surface Modified Upconversion Nanoparticles for an Initial *in Vivo* Molecular Imaging Proof of Principle Approach

**DOI:** 10.3390/ijms13044153

**Published:** 2012-03-29

**Authors:** Anja Hischemöller, Claudia Walter, Volker Weiler, Helga Hummel, Theo Thepen, Michael Huhn, Stephan Barth, Werner Hoheisel, Karen Köhler, Diana Dimova-Landen, Christoph Bremer, Markus Haase, Jens Waldeck

**Affiliations:** 1 Department of Chemistry, University of Osnabrück, Barbarastr. 7, 46069 Osnabrück, Germany; E-Mails: ahischem@uos.de (A.H.); clwalter@uos.de (C.W.); markus.haase@uos.de (M.H.); 2 Philips Research Laboratories Aachen, Weisshausstr. 2, 52066 Aachen, Germany; E-Mails: v.weiler@philips.com (V.W.); helga.hummel@philips.com (H.H.); 3 Frauenhofer IME, Fockenbeckstr. 6, 52074 Aachen, Germany; E-Mails: theo.thepen@ime.fraunhofer.de (T.T.); huhn@molbiotech.rwth-aachen.de (M.H.); barth@molbiotech.rwth-aachen.de (S.B.); 4 Bayer Technology Services GmbH, Building E41, 51368 Leverkusen, Germany; E-Mails: werner.hoheisel@bayer.com (W.H.); karen.koehler@bayer.com (K.K.); dianadimova.landen@bayer.com (D.D.-L.); 5 Molecular Imaging Group, Department of Clinical Radiology, University Hospital Münster, Waldeyerstr. 1, 48149 Münster, Germany; E-Mail: bremerc@uni-muenster.de

**Keywords:** anti-MUC-1 single-chain antibody fragment, upconversion nanoparticles, labeling, *in vivo* optical molecular imaging

## Abstract

*In vivo* optical Imaging is an inexpensive and highly sensitive modality to investigate and follow up diseases like breast cancer. However, fluorescence labels and specific tracers are still works in progress to bring this promising modality into the clinical day-to-day use. In this study an anti-MUC-1 binding single-chain antibody fragment was screened, produced and afterwards labeled with newly designed and surface modified NaYF_4_:Yb,Er upconversion nanoparticles as fluorescence reporter constructs. The MUC-1 binding of the conjugate was examined *in vitro* and *in vivo* using modified state-of-the-art small animal Imaging equipment. Binding of the newly generated upconversion nanoparticle based probe to MUC-1 positive cells was clearly shown via laser scanning microscopy and in an initial proof of principal small animal optical imaging approach.

## 1. Introduction

There is a great demand for new fluorescent labeling materials due to the rapid advantages in monitoring and diagnose of diseases, and thus the development of more efficient and ultrasensitive fluorescent labels is becoming a forceful trend. In addition, optical molecular imaging is a desirable technique for non-invasive *in vivo* diagnostics and follow-up, since it is a highly sensitive, inexpensive imaging method that can potentially resolve relevant target structures *in vivo* [1]. With the injection of a fluorescent reporter—also named probe—optical imaging allows specific tagging of, e.g., receptors, antigens, genes, or drugs, and thereby leadings to a better understanding of molecular disease processes [1–4]. Addressing overexpressed gene products like aminopeptidase *N*-α-V-β-3 or MUC-1 in (breast) cancer tumor cells is highly recommendable for optical imaging approaches, since these are also prognostic markers [5–9]. Furthermore, MUC-1 expression correlates with cancer severity and is inversely correlated to patients’ survival prognosis [6].

Apart from the today well-known band-emitting quantum dot systems [10–12], the use of upconversion particles has attracted the attention of several research groups. In this case, the luminescence depends on dopant ions of the rare earth elements occupying specific lattice sites of the material. A further benefit of such systems is the absence of well-known toxic elements like cadmium and selenium [8,13,14] as well as not suffering from high photobleaching rate or being vulnerable to metabolic or chemical degradation [15]. A couple of *in vitro* assays have been described [16,17] and furthermore some initial *in vivo* tests are known [9,18]. Upconversion nanoparticles convert near infrared radiation (NIR) into visible emission by a multiphoton absorption process, which involves metastable excited states only. Since the lifetime of these metastable states is in the range of microseconds, the two or more NIR photons required for the excitation of the emitting state can be absorbed sequentially rather than simultaneously. Excitation in the NIR at low excitation densities provides some significant advantages compared to excitation in the UV/visible spectral range. For instance, any luminescence excited in the sample by the NIR light will be emitted at a longer wavelength and therefore not in the visible range. This strongly reduces the autofluorescence background, in biological materials leading to a simplified detection of the emitted light and an increased sensitivity [19]. Furthermore, there is no need for time-resolved detection due to the large wavelength separation between excitation and emission. Moreover, excitation in the NIR decreases photo degradation of the biomaterials and may simplify *in vivo* imaging because of the ability of NIR radiation to penetrate more deeply into biological tissue. The emission of an upconversion material depends on the choice of rare-earth ion doped into the crystal lattice of the material and on local properties of the respective lattice sites. In contrast to quantum dots the optical properties do therefore not depend on the particle size except that dopant ions in surface sites usually display lower quantum yield and different crystal splitting of their emission lines. Highly efficient upconversion processes are observed only in host materials with low photon energies, *i.e.*, for materials where the probability for non-radiative multiphonon relaxation processes is low. The best host material for upconversion known today is NaYF_4_ doped with the ion couples Yb^3+^/Er^3+^ or Yb^3+^/Tm^3+^ [20]. The synthesis of nanocrystals of these materials has therefore attracted a great deal of interest and a variety of methods have been developed [21–23]. In these materials, the NIR excitation light of 974 nm is absorbed mainly by the Yb^3+^-ions and subsequently transferred to the co-dopant. In the case of co-doping with trivalent erbium, the energy of two excited Yb^3+^-states is transferred to one Er^3+^-ion, resulting in emission mainly in the green and the red spectral range. Especially the red emission is beneficial for *in vivo* optical imaging; thus a single-chain Fv fragment targeting the MUC-1 receptor was labeled to surface modified upconversion nanoparticles and used for fluorescence optical *in vitro* and a proof of concept *in vivo* molecular imaging approach.

## 2. Results and Discussion

**Figure 1:**
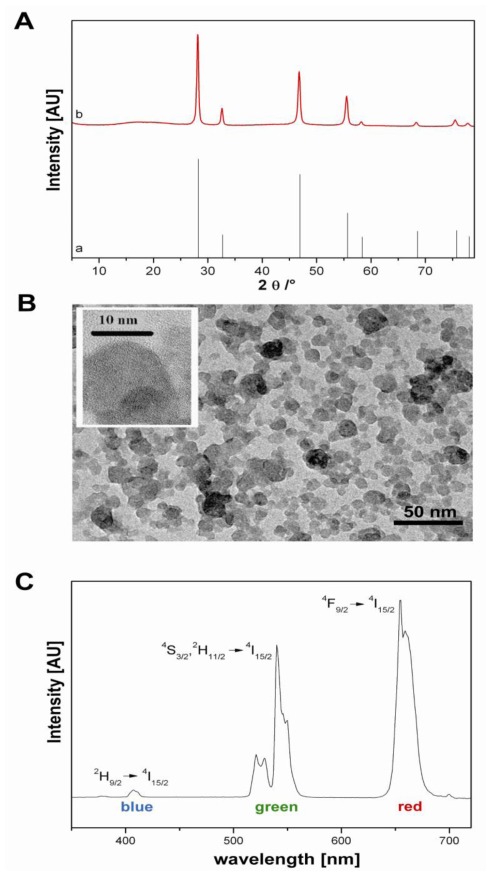
(**A**) X-ray powder diffraction pattern of NaYF_4_:Yb,Er (a; black line) with the corresponding JCPDS No. 77-2042 line pattern for α-NaYF_4_ (b; red line). (**B**) Transmission electronic micrograph of the unconjugated, un-modified upconverting nanoparticles. Scale bars are indicated in the picture. (**C**) Photoluminescence spectra of the upconversion nanoparticles in aqueous colloidal solution (excitation with a 974 nm laser).

The NaYF_4_:Yb,Er-nanocrystals used in this study were synthesized in the coordinating solvent 2-hydroxyethyl-ethylenediamine [5]. The X-ray diffraction data of nanocrystal powders indicate that the nanoparticles crystallize in the cubic α-phase (Figure 1A). From the peak width a mean particle size of 25 nm was calculated with the Debye-Scherrer equation. This was according to the TEM images of the particles displaying a rather broad particle size distribution (Figure 1B). As usual for Yb^3+^/Er^3+^-doped upconversion materials, the emission bands are located at about 405 nm (blue), 550 nm (green) and 660 nm (red) upon excitation at 974 nm (Figure 1C). The appropriate transitions are given in the Figure 1C. The intensity of the blue emission band is rather low, since the emitting level is populated by a sequential three photon step.

**Figure 2:**
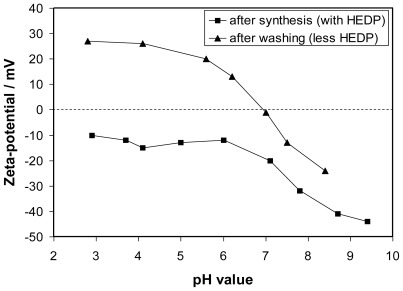
Zeta-Potential versus pH for aqueous dispersions of upconversion nanoparticles after synthesis (solid squares) and after washing (solid triangles) to remove HEDP.

After synthesis the surface of the particles had to be modified in order to allow the attachment of the anti-MUC-1-single chain Fv antibody fragment (M12) as well as to ensure a stable dispersion of these nanoparticles in biocompatible media, e.g., PBS buffer. Since a covalent binding is less suitable for NaYF_4_ surfaces, secondary interactions had to be taken into account for the attachment of the linker. After synthesis the particles were stabilized in aqueous dispersion by the negatively charged HEDP [24]. This low-molecular weight compound is only loosely attached to the particle surface. Zeta-potential titrations of particle dispersions showed that the surface charge had shifted to positive values after washing with water pointing to a removal of HEDP (Figure 2). Nevertheless, electrostatic interactions can be utilized to bind oppositely charged molecules to the particle surface if the molecule possesses a multitude of likely charged centers. Coating of the washed positively charged upconversion nanoparticles (ucNPs) with polyacrylic acid (PAA) of medium molecular weight via electrostatic interaction renders the surface hydrophilic enough to stabilize the particles in PBS buffer as also recently published elsewere [25–27]. Furthermore, PAA provides carboxylic groups [28] to allow the coupling of proteins or peptides via standard EDC/sulfo-NHS reaction. The strong attachment of PAA to the ucNPs was proven by measurements of the electrophoretic mobility. The zeta-potential changes from +20 mV (pH 5) for the washed uncoated particles to −32 mV (pH 5) after PAA coating and subsequent thorough washing. TEM measurements verify that the particles do not agglomerate during polymer coating (data not shown). However, the polymer coating can hardly be seen in the TEM images. Statistical analysis on particle size revealed a mean diameter of 34 ± 11 nm. Similar to the results obtained with uncoated particles (see also Figure 1C) the emitted light of the 1.9% PAA-surface-modified particles exhibited a very low green-to-red ratio, *i.e.*, a much higher intensity of the emission band centered at about 670 nm compared to that at about 550 nm.

**Figure 3:**
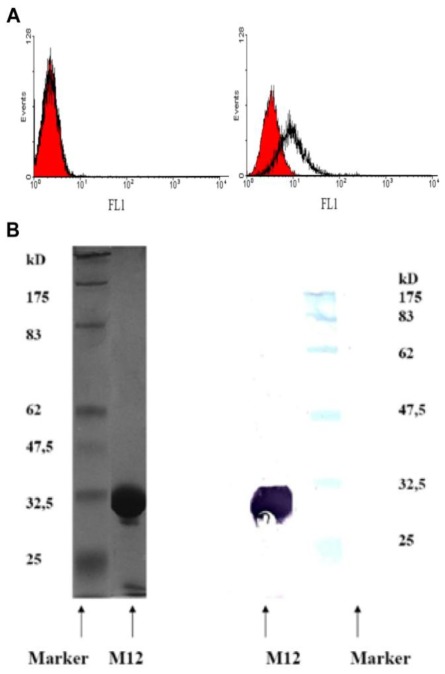
Characterization of the recombinant single-chain Fv fragment M12. (**A**) Binding properties of the anti-MUC-1 M12 to cells by flow cytometry. Cells were incubated with purified M12-scFv (transparent curves) or with PBS as negative control (red curves). No binding was detected at the MUC-1 negative cell line (left) while MUC-1 expressing cell line MDA-MB-231 showed clear binding of the M12-scFv (right). (**B**) Molecular size chromatography sample of the recombinant single-chain Fv fragment M12 documented by SDS-PAGE (left) and immuno-staining (Western blot, right), immunodetection: 1st antibody: anti-His (Qiagen AG, Germany); 2nd antibody: alkaline-phosphate-conjugated anti mouse-IgG moab 3. Staining: substrate 5-bromo-4-chloro-3 indoyl-phosphate (BCIP) was used in conjugation with the enhancer, nitro blue tetrazolium (NBT).

**Figure 4:**
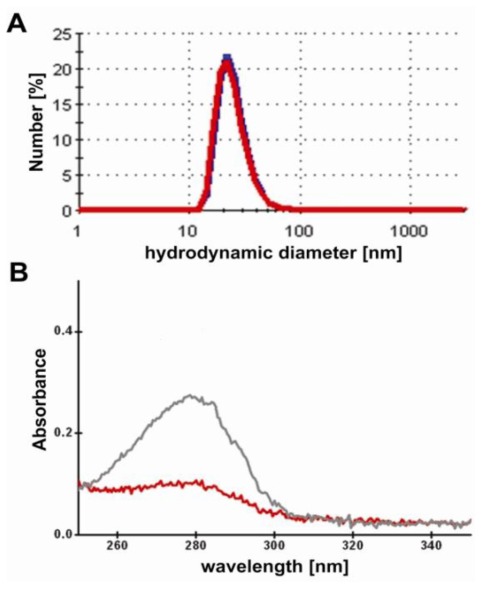
(**A**) Hydrodynamic diameter of the conjugates (red) and the unconjugated upconversion nanoparticle reference (blue) after 100 kDa-purification, both in PBS (pH 7.4). (**B**) Absorption spectra of the labeling mixture (M12-ucNP; red) and M12-reference (M12; grey) after 100 kDa-purification.

The PAA-modified NaYF_4_:Yb,Er particles were then labeled to the anti-MUC-1-single chain Fv antibody fragment (M12) for fluorescence imaging. The used M12 was produced as described in the Experimental Section with a purification rate of >95% as shown in both SDS page as well as Western blot analyses (see also Figure 3B), which reflects the purification rates obtained elsewhere [21]. The EDC-mediated labeling reaction result in M12-ucNP conjugates showing the respective blue, green and red fluorescence spectra with similar intensities compared to the original and PAA-modified upconversion nanparticel (ucNP) solution. Obviously, the ucNPs do not agglomerate during the labeling reaction as visible in Figure 4A, showing the hydrodynamic diameter of the conjugates and non-labeled ucNP reference particles. The conjugates are labeled with about 15 units M12 single-chain Fv fragments per particle. This was roughly estimated from the specific M12-absorption at 280 nm (ɛ_M12_~44.3 kM^−1^cm^−1^) using the 100 k-filtrates of the conjugates and the unlabeled M12 reference (see Figure 4B).

The underglycosylated MUC-1 receptor is one of the key-players in breast cancer formation and thus highly overexpressed [29,30]. To validate the synthesis of the MUC-1 receptor different human cancer cells had to be checked: The MDA-MB-231 and BT-20 breast cancer cell lines exhibited high MUC-1 expression while HT-1080 showed no detectable MUC-1 production (see Material and Methods). In addition, cell binding studies by FACS showed specific binding to the MDA cells expressing MUC-1, whereas the non-expressing control cell line showed no binding (see Figure 3A). Thus, MDA-MB-231 cells were chosen as positive while HT-1080 cells served as negative controls.

**Figure 5:**
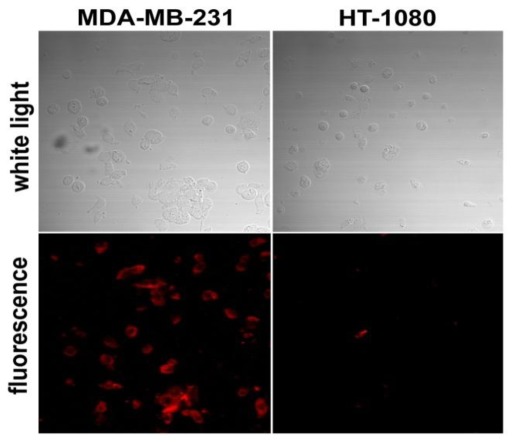
Laser scanning microscopy of MDA-MB-231 and HT-1080 cells after incubation with anti-MUC-1-ucNP conjugates (20×). Binding to MDA-MB-231 was visible in the fluorescence channel while no binding to HT-1080 could be observed.

A distinct binding of the M12-ucNP construct to the MUC-1 receptor exhibiting cells was shown in microscopic studies (Figure 5). Upconversion nanoparticles (ucNP) without targeting units (*i.e.*, without M12) neither bound to MDA-MB-231 nor HT-1080 cells, respectively (data not shown). In addition, nearby no binding to MUC-1 receptor negative HT-1080 cells was observed (Figure 5), thus showing the specificity of M12-ucNP binding to only MUC-1 receptor overexpressing cells. Those findings are congruent to recently published results with PAA-modified ucNP from Jin *et al*. [25] where a highly reduced cell uptake was observed after PAA treatment. Xiong *et al*. [8] and Zako *et al*. [9] both developed upconversion particles that were labeled to cyclic RGD peptides addressing tumor-derived α-V-β-3 overexpression. In their microscopic experiments the specific binding was also shown.

**Figure 6:**
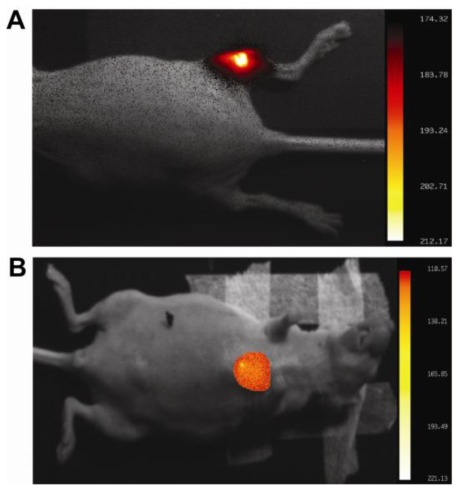
*In vivo* optical molecular imaging proof-of-principal approach in nude mice. Intensities are given in arbitrary units (AU). Imaging parameters were 300 s, f/2.5, 30 mW 978 nm laser light source, 850 cut of NIR filter in an modified MS FX PRO small animal *in vivo* imaging system (Carestream Molecular Imaging). (**A**) Fluorescence imaging of unlabeled upconversion nanoparticles (ucNP) injected into the muscle of a mouse leg. (**B**) Anti-MUC-1-ucNP based signal of the MDA-MB-231 xenograft. The fluorescence signal was pseudo-colored and overlayed to a white light reference image.

To check the ucNPs’ suitability for near infrared *in vivo* optical imaging unlabeled and M12 labeled ucNPs were injected intramuscular into nude mice and imaged. In both cases, a clear fluorescence signal could be observed (300 s, f-stop 2.5, Figure 6A). Based on those results MDA-MB-231 adenocarcinoma cells were implanted into a nude mouse and subjected to *in vivo* imaging. Binding of the M12-ucNP to the MUC-1 receptor expressed by the MDA-MB-231 tumor cells resulted in a fluorescent signal while the surrounding tissue was non-fluorescent (Figure 6B) likewise observed by Yu *et al*. [31] when using a chlorotoxin-labeled upconversion nanoparticle for tumor imaging. However, the high energy-input and time that was required for imaging in our case resulted later in an actinocutitis which was comparable to a sunburn. This observed actinocutitis based on the time-based high-energy input (300 s, 30 mW at 978 nm constant laser light) and also the high concentrations of probe needed (1 mM) still shows the limitations that are based on (i) the recently available instrumentation as well as the (ii) the used M12-ucNP probe design. Using microscopic approaches on transparent HeLa cells resulted in a higher signal-to-noise ratio when increase the excitation power [32]. In our approach, the use of light pulses with pulse durations in the microsecond to nanocecond range instead of continuous wave excitation is supposed to reduce the energy input but not violate the fluorescence signal and thus might reduce the negative side effects dramatically by also increasing the signal-to-noise ratio. Also the use of high resolution, cooled, back-illuminated CCD cameras with a high quantum efficiency (QE) like the recently available 4 MP back-illuminated Xtreme camera system that provide a QE of >95% (Carestream Molecular Imaging) or by use of modern EMCCDs [8] should increase the detection limit and thus avoid the observed side-effects. Further developments of the ucNPs regarding their fluorescence yield, e.g., by using ucNP with a non-doped shell (core-shell nanoparticles) may also increase the signal-to-noise ratio and thus allow shorter imaging times as well lower probe concentrations. Increasing the red-to-green ratio of the emission might further improve that approach.

However, the developed approach is of use because patients who are at high risk to develop, e.g., breast cancer [33] express the MUC-1 antigen at an early stage. Thus, early cancer detection, staging and treatment-follow up via imaging is highly beneficial and had been shown previously with a dual labeled MRI/fluorescence probe [34]. The benefit of using upconversion nanparticles *versus* classical, e.g., Cyanin dyes is based on the used excitation light that should penetrate the tissue with less absorption and thus allow deeper tissue imaging in the future.

## 3. Experimental Section

### 3.1. Synthesis of Upconversion Nanoparticles

Water soluble a-phase NaYF_4_:Yb,Er (20 mol% Yb, 2 mol% Er) nanoparticles were synthesized and surface modified with HEDP (hydroxyethyl-diphosphonic acid) according to a published procedure in the coordinating solvent *N*-(2-hydroxyethyl)ethylendiamine (HEEDA) [35].

### 3.2. Surface-Modification of Upconversion Nanoparticles

For Polyacrylic acid (PAA) coating the nanoparticles stabilized with HEDP were first thoroughly washed with water to remove the excess of the organophosphoric stabilizer using centrifugation. Afterwards the suspension was diluted with aqueous HCl solution until a final upconversion nanoparticle (in short: ucNP) concentration of 0.05% (w/w) and a pH of 2.3 was reached. One hundred millilitres of this nanophosphor suspension were added to 100 mL 0.5% aqueous solution of NaPAA (pH 9.3, Sigma-Aldrich) and stirred at room temperature for one hour. To remove the unbound PAA the nanoparticles were thoroughly washed with water using a stirred ultrafiltration cell equipped with a regenerated cellulose membrane (cut-off 100 kDa, Vivascience, Hannover, Germany).

### 3.3. Particle Characterization

For TEM measurements the dispersion was dried on a carbon-coated copper grid and investigated by means of a Philips Tecnai 20 transmission electron microscope operating at 200 kV. Up-conversion emission spectra of ucNP dispersions were measured with a Fluorolog 3 FL3-22 spectrometer (Horiba Jobin Yvon GmbH, Bensheim, Germany) combined with a 200 mW IR laser module (Roithner Lasertechnik GmbH, Vienna, Austria). Quartz cuvettes containing the samples were placed inside the spectrometer and excited by the 980 nm light of the laser.

Electrophoretic mobility of ucNPs was measured in dilute aqueous dispersions (0.05% (w/w)) by means of 90 Plus and the appendant zeta-potential module (Brookhaven Instruments Limited, Worcestershire, UK). The pH of the dispersions was carefully adjusted with HCl and NaOH solution, respectively.

### 3.4. Production of Anti-MUC-1 Single-Chain Fv Fragments

#### 3.4.1. Propagation of Phage from the Human Single-Chain Fv Phage Display Library

One liter of 2× TY, 100 μg/mL ampicillin, 1% (w/v) glucose was inoculated with an aliquobrary 10^9^ diversity glycerol stock. The rescue of the phage was carried out as described in [36].

#### 3.4.2. Selection of Human Single-Chain Fv Fragment

Human single-chain Fv antibody fragments against the cancer antigen MUC-1 were selected directly on the human breast cancer cell MCF-7 (ATCC: HTB-22). After three rounds of selection, the individual bacterial clones were induced to produce soluble anti-MUC-1 single-chain Fv fragments (in short: M12). These single-chain Fv fragments were tested on ELISA for their ability to bind to the MCF-7 cell line and the MUC-1 core protein [36].

### 3.5. Fermentation of Single-Chain Fv Fragments (M12)

Immunoglobulin variable-region genes (VH and VL) were PCR amplified from 5 mL of cDNA, assembled, and cloned into pCANTAB6 as described elsewhere [36]. Plasmids were transformed into 50 μL *Escherichia coli* TG-1 by electroporation [37]. Fermentations of anti-MUC-1 single-chain Fv fragments (M12) were carried out in a 7 L working volume stirred tank bioreactor (biobench 7, Applikon, Schiedam, The Netherlands) using a synthetic mineral medium (16.6 g/L KHPO_4_, 4 g/L NH_4_(H_2_PO_4_), 0.07 g/L CaCl_2_·2H_2_O, 0.15 g/L FeSO_4_·7H_2_O, 1.5 g/L MgSO_4_·7H_2_O, 2.1 g/L citric acid, 0.2 g/L l-arginine, 0.2 g/L l-methionine, 25 mg/L Kanamycin and 25 g/L glucose supplemented with 1 mL/L of Ptm1 trace salts (Invitrogen, Darmstadt, Germany, pH 6.8) at 30 °C and constant aeration at 1 L^−1^·min^−1^. Fermentations were inoculated with 250 mL of a preculture in LB medium grown to an OD of 1.5. When the initial amount of glucose was exhausted, glucose was added using the dO_2_ signal to control the glucose feed rate, until an OD of 20 was reached. At this time, the M12-expression was induced by the addition of 0.5 mM IPTG and temperature was lowered to 28 °C. Induction was carried out for 24 h until the fermentation was harvested. The M12 single-chain Fv fragments were expressed into the periplasm of the *E. coli* strain and afterwards purified by Ni-NTA chromatography.

### 3.6. Purification of M12 Single-Chain Fv Fragments

For Ni-NTA affinity purification of M12 fragments, a Streamline 25 column (Pharmacia/GE Healthcare, Freiburg, Germany), packed with 100 mL of Streamline Chelating medium (Pharmacia), was charged with 3 times the column volume of 50 mM NiSO4 and equilibrated with 10 column volume of binding buffer (PBS containing 1 M NaCl, pH 8). Bulk cell mass was separated from the fermentation supernatant by centrifugation (10 min, 10,000 g). A 1/9 volume of 10× PBS and 50 g/L NaCl was added to a final concentration of 1 M NaCl, pH was adjusted to 8, and the conditioned fermentation broth was passed over the column in expanded mode at a flow rate of 410 cm/h. The column was then washed in expanded mode with binding buffer until the flow through was particle-free. Then the flow was reversed, the flow rate set to 100 cm/h and a 5-column volumes wash with binding buffer supplemented with 10 mM imidazole was performed in packed-bed mode. Bound M12 fragments were eluted with binding buffer containing 250 mM imidazole and the protein-containing eluate, determined by monitoring the A280, was collected.

The M12 single-chain Fv fragments were purified by ion exchange (anion exchange) chromatography using MonoS Sepharose (GE Healthcare) on an Äkta FPLC system (GE Healthcare). The M12 fragments were eluted with Na-acetate (pH 4.5) and a linear gradient 20–1000 mM NaCl. Final polishing and endotoxin removal were performed by size exclusion chromatography (SEC) with Sepharose 200/300 (GE Healthcare). Recombinant M12 fragments were eluted with PBS (pH 7.4) at a flow rate of 1 mL/min. Samples were fractionated and stored for further analysis at 4 °C.

SDS-PAGE and Western blotting were performed to determine the M12 fragment quality [38]. M12 fragments were detected by anti-His mAb (Qiagen, Hilden, Germany). Bound antibody was stained with an alkalinephosphatase-conjugated anti-mouse-IgG monoclonal antibody (mAb, Sigma-Aldrich, St Louis, USA) and a solution of Tris-HCl (pH 8.0) and 0.2 mg/mL naphtol-AS-Bi-phosphate (Sigma-Aldrich) supplemented with 1 mg/mL Fast-Red (Serva, Heidelberg, Germany).

### 3.7. Flow Cytometric Binding Analyses

Cell-binding activity of M12 fragments was evaluated using a FACSCalibur flow cytometer and CellQuest software (Becton Dickinson, Heidelberg, Germany). Cells were stained with the affinity purified M12 fragment as described previously. Briefly, 10,000 events were collected for each sample, and analyses of intact cells were performed using appropriate scatter gates to exclude cellular debris and aggregates. A total of 5 × 10^5^ cells were incubated for 1 h on ice with 50 μL of the M12-bacterial protein extract at a concentration of 30–40 μg/mL. The cells were washed with PBS buffer containing 0.2% (w/v) BSA and 0.05% (w/v) sodium azide and then incubated for 30 min with an anti-His mAb diluted 1:2 in PBS buffer. Cells were washed and incubated with FITC-labeled goat-anti-mouse IgG (Dako Diagnostica, Hamburg, Germany) for 1 h at 4 °C. After a final wash, the cells were treated with 2 μL of 6.25 mg/mL Propidium Iodid and subsequently analyzed by fluorescence-activated cell sorting [38].

### 3.8. Labeling of Upconversion Nanoparticles with Anti-MUC-1 Single-Chain Fv Antibody Fragments

The anti-MUC-1 single-chain Fv antibody fragments (M12) were attached to PAA-modified upconversion nanoparticles using the popular cross-linker carbodiimide EDC 1-ethyl-3-(3-dimethylaminopropyl) carbodiimide (Sigma-Aldrich, St Louis, USA). EDC mediates the formation of amide linkage between carboxylate-equipped ucNPs and amine groups found at the M12 single-chain Fv fragments. The reaction was performed in a 2 mL Eppendorf-tube by adding 450 μL ucNP suspension (approx. 0.2 μM, measured by UV), 82 μL M12 solution (approx. 22 μM) and 70 μL PBS (pH 7.4, Sigma-Aldrich). After mixing the educts, 23 μL EDC (10 mM) are rapidly added to the stirred solution. In this case, the carboxylate groups were activated with EDC resulting in the active intermediate *O*-acylisourea. Subsequently, the intermediate can react with amine groups present in the reaction mixture. After mixing the solution for 1.5 h, the conjugates were purified by ultrafiltration (cut-off 100 kDa, Vivascience) to remove excess reagent, by-products and non-bound M12 (MW~28 kDa). Finally, the conjugates (shortly named: M12-ucNP) were re-dispersed in PBS (pH 7.4). As references, the M12 single-chain Fv fragments and the ucNP were treated in the same manner as applied in the labeling reaction, but without the mediator EDC. The number of M12 single-chain Fv fragments bound to the particles is roughly estimated from the specific M12-absorption at 280 nm (ɛ_M12_~44.3 kM^−1^cm^−1^) using the 100 k-filtrates of the conjugates and the unlabeled M12 reference. The intensity of the absorption at 280 nm was measured with a commercial spectrometer from Perkin Elmer (lambda9). The concentration of the M12 single-chain Fv fragments was calculated from the absorption and the extinction coefficient according to Lambert-Beer’s law.

### 3.9. MUC-1 Expression Profiles of Cancer Cell Lines

Cell lines were cultured under standard conditions according to the suppliers’ recommendations (AATC protocols, LGC Standards GmbH, Wesel, Germany). Western blotting was performed as described elsewhere [5]. Shortly, a MUC-1 specific antibody (C595 Mouse anti Human MUC-1 Antibody, AbD Serotec, Duesseldorf, Germany; 1 μg/μL) was used to check the expression of the MUC-1 antigen of MDA-MB-231 (ATCC: HTB-26), DU4475 (ATCC: HTB-123), BT-20 (ATCC: HTB-19), MCF-7 (ATCC: HTB-22), SK-BR-3 (ATCC: HTB-30), HT-1080 (ATCC: CCL-121) and MDA-MB-435 (ATCC: HTB-129) cell lines, respectively. Results were validated via semi-quantitative RT-PCR using the forward primer MUC-1-for (GTG CCC CCT AGC AGT ACC G) and the reverse primer MUC-1-rev (GAC GTG CCC CTA CAA GTT GG; Eurofins MWG Operon, Ebersberg, Germany). The annealing was conducted at 61 °C followed by an elongation step at 72 °C using TripleMaster DNA-Polymerase (Eppendorf AG, Hamburg, Germany) with 35 repetition steps. DNA gel electrophoresis was performed using a 1.5% (w/v) agarose gel and bands were captured with the Gel Logic 200 system (Carestream Molecular Imaging, Carestream Health Inc, Woodbridge, CT, USA).

### 3.10. *In Vitro* Binding Studies

Binding of the M12-ucNP conjugate probe to selected cancer cell lines (HT-1080, MDA-MB-231 and MDA-MB-435) was checked via laser scanning microscopy (Zeiss LSM 510 Meta Carl Zeiss Jena GmbH, Jena, Germany) combined with the Ti:Sapphire Laser Chameleon-Ultra by Coherent Deutschland GmbH (Dieburg, Germany). The laser excitation wavelength was 974 nm, the laser pulse width 140 femtoseconds, with a repetition rate of 80 MHz and the power at 974 nm was about 0.8 W. 1 × 10^6^ cells were carefully washed with PBS buffer solution (pH 7.2, 0.01% Tween 20) and incubated in PBS/BSA (pH 7.2, 1% BSA) for 1 h on ice. After washing with PBS the cells were incubated for 1 h with M12-ucNPs probe and non-labeled ucNPs, respectively. Subsequently, cells were washed and imaged.

### 3.11. Tumor Xenografts

All animal studies were approved by the institutional review boards (# G76/2005). Female athymic nude mice (*nu*/*nu)* were obtained from Charles River Laboratories (Erkrath, Germany). At 4–6 weeks of age, 3 × 10^6^ MDA-MB-231 cells suspended in 100 μL PBS were injected subcutaneously into the mama fat pad. When tumors reached 4–6 mm in diameter, the tumor-bearing mice were anaesthetized by *i.p.* injection of ketamine (125 mg/kg body weight) and xylazine (12.5 mg/kg bw) and subjected to *in vivo* imaging studies.

### 3.12. *In Vivo* Fluorescence Reflectance Imaging

Fluorescence images were acquired using the modified small animal *in vivo* Imaging Station MS FX PRO (Carestream Molecular Imaging/Carestream Health, Woodbride, CT, USA). The Xenon lamp was replaced by a continuous wave 978 nm laser diode (LYPE 30-SG-WL978-F400 by Roithner, Austria). A 850 nm cut off filter (Roithner, Austria) in front of the optics was used to block near infrared wavelength. Non-conjugated ucNP were injected intramuscularly into mice to check their imaging abilities (final concentration 0.1 μM). M12-ucNPs were injected into MDA-MB-231 bearing nude mice with a final concentration of 1 mM via a 150 μL *i.v.* tail vein injection and images were captured 30 min after injection. Acquisition times were 300 s at f-stop 2.5.

## 4. Conclusions

In this proof of principle investigation, an anti-MUC-1 single-chain Fv fragment was produced, purified and labeled to newly designed surface modified upconversion nanoparticles for *in vitro* and *in vivo* imaging approaches. The binding of the M12-ucNP probe could be verified *in vitro* as well as in an initial experiment *in vivo*. Tumor signal accumulation of M12-ucNPs in a xenograft model showed the feasibility of this approach but demonstrate the need for further improvements on the instrumentation as well the probe and fluorophore side.
